# Social connection and sociodemographic predictors of stroke caregivers' burden during COVID-19 pandemic lockdown

**DOI:** 10.3389/frhs.2026.1901816

**Published:** 2026-08-12

**Authors:** Muhamad Faizzuddin Razali, Shamala Ramasamy, Shue Ling Chong, Po Ling Chen

**Affiliations:** 1 Department of Chiropractic, School of Alternative and Complementary Medicine, IMU University, Kuala Lumpur, Malaysia; 2 Department of Psychology, School of Psychology and Social Services, IMU University, Kuala Lumpur, Malaysia; 3 School of Psychology, Faculty of Science and Engineering, Semenyih, Selangor, Malaysia; 4 School of Health and Life Sciences, University of Nottingham Ningbo, Ningbo, China

**Keywords:** caregiving burden, corona virus, healthcare, informal caregivers, social restriction

## Abstract

**Introduction:**

Stroke care demands significant effort and is heavily reliant on physical rehabilitation facilities to improve the patients' functional status. Limited access to these facilities during COVID-19 lockdown impacts patients' recovery and family caregivers' burden. The impact of COVID-19 lockdown on caregivers' duties and its potential negative effects on family caregivers' burden remained scarce. This study examined the social connections' predictors and stroke caregivers' burden during COVID-19 lockdown.

**Methods:**

This study employed a cross-sectional predictive study design and 94 unpaid informal family caregivers of stroke survivors completed an online survey. The caregivers' burden was measured using the Caregiver Burden Inventory. Social connection such as social isolation, loneliness and perceived social support was measured using the Lubben Social Network Scale, Loneliness Scale and Duke Social Support Index, respectively. Sociodemographic data were also collected and analysed.

**Results:**

Multiple linear regression analysis revealed that social support significantly predicted the stroke caregivers' burden. Social support had the major predictive effect, explaining 12% of the variance in caregivers' burden. Meanwhile, loneliness significantly predicted developmental burden and physical burden respectively. Social isolation did not significantly predict caregivers' burden. Caregivers with tertiary education reported significantly higher burden than caregivers with secondary education. Education was also a significant predictor of developmental burden, physical burden, and emotional burden.

**Discussion:**

These findings highlight that family caregivers' burden during COVID-19 lockdown is linked to their social support, loneliness, and education levels. Interventions that aim to enhance social support for caregivers could help to mitigate this issue and improve their overall wellbeing.

## 1 Introduction

Stroke survivors often experience long-term impairment and functional dependency, putting considerable demands on individuals, families, and healthcare resources (1). Consequently, many rely on family members, relatives, friends, or neighbours—collectively referred to as informal caregivers—for ongoing support and assistance with daily activities (2). The emergence of the corona virus disease 2019 (COVID-19) pandemic further intensified these challenges by disrupting healthcare services worldwide. In particular, lockdown measures and restrictions on movements limited access to rehabilitation services and therapy centres, reducing the availability of formal support and increasing reliance on informal care (3). Consequently, caregivers were required to undertake additional caregiving tasks and responsibilities, leading to an escalation in caregiver burden (4). This elevated burden manifested across multiple dimensions, including physical, emotional, social, developmental burden, and time-related burden (4, 5).

Specifically, the physical burden, manifested as fatigue, headaches, joint pain, and sleep disturbances (6), has been reported to increase following the interruption of formal care services in stroke rehabilitation facilities (7, 8). Beyond the physical demands of caregiving, stroke caregivers often experience emotional burden, including heightened levels of stress, depression, and anxiety associated with the provision of care (9). In Malaysia, the implementation of Movement Control Orders in March 2020, which restricted movement and social interactions to curb the spread of COVID-19 (10), likely increased caregivers' emotional burden due to reduced access to support services, heightened caregiving responsibilities, and concerns about being blamed by family members for adverse caregiving outcomes (11, 12).

In addition to physical and emotional burden, caregivers often experience developmental burden, which is characterised by feelings of missing out on life opportunities and perceiving caregiving as a constraint on one's personal life (13), and is commonly reported by caregivers within the first three months after stroke onset (14). As caregiving responsibilities intensity, stroke caregivers are often required to sacrifice their personal needs and aspirations, such as altering or postponing future plans to accommodate long-term caregiving commitments (15). These substantial lifestyle adjustments can compromise their mental health, social relationships, and overall wellbeing, thereby contributing to increased developmental burden.

Furthermore, stroke caregivers may also experience substantial social burden, which refers to the strain arising from disruptions in social relationships, social participation, and diminished access to support networks as a result from the caregiving responsibilities. In this study, social connectedness among stroke caregivers was reflected through their perceived level of social support, sense of isolation, and feelings of loneliness. Disruptions to these social connections may adversely affect caregivers' wellbeing and contribute to greater social burden (16). During the lockdown, movement restrictions and social distancing measures limited opportunities for caregivers to share their physical and developmental burdens with others who are residing in different locations (7). Consequently, the prolonged long hours of caregiving, coupled with reduced social interaction and support likely intensified feelings of isolation and loneliness, thereby exacerbating the social burden experienced by stroke caregivers during the pandemic.

Social isolation and loneliness, which represent the objective and subjective dimensions of social disconnection, respectively (17), are distinct constructs. Specifically, social isolation denotes an objective lack or limited extent of social contacts and social engagement, whereas loneliness refers to the subjective perception of social isolation and the emotional experience of feeling alone (18). The pandemic exacerbated pre-existing vulnerability to social isolation and loneliness among elderly stroke caregivers (19). Social isolation due to the lockdown has disrupted caregivers' support networks from family and the wider community. Compared with the pre-pandemic period, informal caregivers also reported significant higher levels of caregiving burden, social isolation, and loneliness (20). These findings underscore the complex and multifaceted challenges faced by informal stroke caregivers, whose responsibilities are often compounded by reduced social support and limited opportunities for social engagement.

Social support is an essential resource for stroke caregivers, who normally seek support from the local community, including relatives, neighbours, and household members (6, 21). Although caregiving can be a source of stress, social support has been shown to buffer its adverse effects and promote caregiver wellbeing (22, 23). Its importance became particularly evident during the COVID-19 pandemic, as utilisation of telephone support service and community-based support increased among stroke caregivers and survivors between 2020 and 2021 (24).

In terms of sociodemographic factors, following COVID-19 lockdown, families with low socioeconomic (SES) for instance, were disproportionately affected due to lack of access to healthcare, experiencing higher rates of COVID-19 infection, morbidity, and mortality, thus carrying significant burden (25, 26). Lower SES stroke caregivers often struggle to afford private rehabilitation services, which could put on extra burden to both their existing daily household chores and caregiving duties (27, 28). In contrast, caregivers with higher SES, particularly those who are employed and with higher incomes, tend to have greater access to social support. This allows them to afford private services, minimising disruptions to their caregiving roles and household chores, ultimately reducing their physical burden (29, 30).

Taken together, the substantial caregiving demands imposed on informal stroke caregivers, coupled with limited opportunities for self-care and a lack of financial compensation, can adversely affect their emotional wellbeing, physical health, and overall quality of life (31). These challenges were likely exacerbated during the COVID-19 pandemic, when disruptions to healthcare services and restricted access to stroke rehabilitation facilities increased caregivers' responsibilities and reduced the availability of formal and informal support. While previous studies have documented heightened caregiver burden during the pandemic (4), less is known about how caregiver's sociodemographic characteristics and social connectedness influence different dimensions of burden. Given the important role of social isolation, loneliness, and social support in shaping caregivers' experiences, a better understanding of these factors is needed to identify caregivers who may be particularly vulnerable to adverse outcomes. Therefore, this study aimed to identify the extent to which sociodemographic characteristics and social connection factors, namely social isolation, loneliness, and social support contribute to the different dimensions of burden among informal stroke caregivers. This study was partially informed by the conceptual framework proposed by Razali et al. (4). Nevertheless, due to its exploratory nature, no specific *a priori* hypotheses were formulated regarding the relative predictive importance of individual social connection variables across the different dimensions of caregiver burden. By identifying factors associated with caregiver burden, this study seeks to improve the development of targeted support strategies to improve caregiver wellbeing and resilience during periods of heightened caregiving demands.

## 2 Methods

### 2.1 Study design

This study employed a cross-sectional predictive study design using a survey. Facebook Stroke Survivors Malaysia group page on social media platform and caregivers from National Stroke Association Malaysia were purposively selected from 21st September 2021 to 31st January 2023. *A priori* G power analysis suggested 200 participants (including additional of 20% to accommodate withdrawals as well as missing data) needed for this study to achieve a moderate effect size (Cohen's *f*^2^ = 0.35) for three predictors in the multiple regression analysis with a 95% chance detecting it.

The survey was conducted online via Google form which comprised of study's description, ethical consideration incorporating risks and benefits, participant's rights, authorisation, administration time and contact information. This project was approved at the International Medical University Joint Committee on Research and Ethics on 24th June 2021 (Project ID: MMHS I/2021(04). All participants provided written informed consent before proceeding with the survey. All information regarding respondents and the data collected are kept private and confidential. The study was conducted in accordance with the Declaration of Helsinki. All participants provided written informed consent before proceeding with the survey which was stored securely on a password-protected Google Drive account. They also were informed of their right to terminate their participation in the research at any point. The questionnaires were available in dual-language (English and Malay).

### 2.2 Participant recruitment

The study recruited 114 participants in total based on the inclusion criteria a) Caregivers who care for household or non-household member who suffered a stroke. b) Caregivers who have functioned as a caregiver for at least one month prior to participating in this study; and c) Caregivers who aged 18 and above and able to communicate in English or Malay. Of the total number of participants, 20 (18%) individuals were excluded from this study as they are caregivers who are currently providing care to other than the stroke-care recipient, or they are being paid as caregivers for institutionalised stroke patients. This left with a total of 94 (82%) individuals included in the final data analysis.

### 2.3 Measures

#### 2.3.1 Demographic and social connection

A demographic questionnaire was developed for this study (see Supplementary File 1). In the current study, the social connection comprised of three separate measures: social isolation, loneliness, and social support. All social connection questionnaires were translated to Malay language based on the original version in English (32–36). The reliability of the Malay and English versions of the questionnaires was assessed using Cronbach's alpha. The results showed that both versions had high reliability for all four scales: LSNS-6, UCLA-3, DSSI-11, and CBI. The Cronbach's alpha coefficients in the current study for the Malay version ranged from .84 to .94, and for the English version from .84 to .97, suggesting strong to excellent internal consistency for both versions of the questionnaires.

#### 2.3.2 Lubben social network scale (LSNS-6)

The LSNS-6 scale measures social interactions with friends and family members and their perceived level of social isolation (32). It consists of six items, administered using a Likert Scale ranging from “None = 0”, “One = 1”, “Two = 2”, “Three or Fou*r* = 3”, “Five to Eight = 4”, and “Nine or more = 5”. The questionnaire measures the frequency and quantity of social interactions, as well as friend's isolation. For examples, “How many relatives do you see or hear from at least once a month?”, “How many relatives do you feel at ease with that you can talk about private matters?”. In addition to family isolation, similar questions were inquired to measure a friend's isolation. For instance, “How many friends do you see or hear from at least once a month?”. The cumulative score ranges from 0 to 30, with a score of 12 or below indicating presence of social isolation (32). The LSNS-6 had previously undergone validation and had a Cronbach's alpha of.81 (33). The questionnaire took about five to ten minutes to complete.

#### 2.3.3 Loneliness scale (UCLA-3)

The UCLA-3 consists of three items that aims to assess subjective experience of loneliness, namely relational connectedness, social connectedness, and self-perceived isolation (34). For examples, “How frequently do you perceive a deficiency in companionship?”, “What is the frequency with which you experience feelings of exclusion?”, “What is the frequency at which an individual experiences a sense of social isolation?”. The questionnaire employs Likert Scale response format of “Hardly eve*r* = 1”, “Some of the time = 2” and “Often = 3”. The summation of the scores allocated to each distinct question can yield a feasible spectrum of scores ranging from 3 to 9. Scores ranging from 3 to 5 deemed as “non-lonely”, while scores between 6 and 9 are classified as “lonely” (34). The scale's reliability was deemed satisfactory, as evidenced by its Cronbach (*α*) value of.72 in a previous study (34). The questionnaire took about three to five minutes to complete.

#### 2.3.4 Duke social support index (DDSI-11)

The DDSI-11 measures social support on the two subscales of social interaction and subjective support (satisfaction) among stroke caregivers (37). The subscale for social interaction comprises a total of four items. These items employed Likert Scale “None = 0”, “Once = 1”, “Twice = 2”, “Three times = 3”, “Four times = 4”, “Five times = 5”, “Six times = 6”, and “Seven or more times = 7”. However, the second, third and fourth items were recoded based on the suggested protocol (35). Sample recoded item includes “How many times during the past week did you spend time with someone who does not live with you, that is, you went to see them, or they came to visit you or you went out together?”. Sample items for subjective social support include “Does it seem that your family and friends (people who are important to you) understand you?” were given the options such as “Hardly eve*r* = 1”, “Some of the time = 2”, and “Most of the time = 3”. Satisfaction on subjective support were also asked, “How satisfied are you with the kinds of relationships you have with your family and friends?” with the following Likert scale ranges from “Very dissatisfied = 1”, “Somewhat dissatisfied = 2”, and “Satisfied = 3”. The DSSI-11's overall score spans from 11 to 33, whereby greater values correspond to elevated levels of assistance (35). The internal consistency of DSSI-11 was validated in a previous study with a Cronbach's alpha of .70 (38). In this study, the internal consistency of the Malay and English version yielded a Cronbach's alpha of 0.84 and 0.88, respectively. The questionnaire took about five to ten minutes to complete. According to the manual of DSSI-11, the Cronbach's alpha of this tool should be done following the changes/re-coded. Hence, the value given in this study is consistent with the DSSI protocol (35).

#### 2.3.5 Caregiver burden inventory (CBI)

The CBI measures the multidimensional aspect of the burden experienced by caregivers (36). The inventory comprises a total of 24 items that are categorised into five distinct domains, namely time, developmental, behaviour, physical, social, and emotional burden, with the use of Likert scale ranges from “Never” = 0, “Rarely” = 1, “Sometimes” = 2, “Quite frequently” = 3, “Nearly always” = 4. Sample items for time burden include “He/she needs my help to perform many daily tasks”, and “He/she is dependent on me”. Sample items for developmental burden include “I feel that I am missing out on life”, and “I wish I could escape from this situation”. Sample items for physical burden include “Caregiving has made me physically sick”, and “I'm physically tired”. Sample items for emotional burden include “I feel embarrassed over his/her behaviour”, and “I feel ashamed of him/her”. Finally, sample items for social burden include “My caregiving efforts aren't appreciated by others in my family”, and “I feel resentful of other relatives who could but do not help”. The current study has established the internal consistencies with the coefficients of .89, .89, .92, .88, and .84 respectively for the time burden, developmental burden, physical burden, emotional, and social burden. The questionnaire took about ten to 15 min to complete.

#### 2.3.6 Supplementary open-ended questions

Moreover, two open-ended questions were posed to deepen the scope of the study (see Supplementary Table 2), aiming to uncover challenges faced by participants during the lockdown and to explore the types of support they wished they would have obtained. The supplementary interview was conducted on a one-to-one basis using an online platform such as Google meet or Microsoft TEAMS for the seven participants who were selected based on their scores on the CBI, with four participants scoring at the higher end (CBI Score = 64 to 78) and three participants scoring at the lower end (CBI Score = 2 to 7) of the CBI spectrum. The additional verbal consent was obtained to participate in the interview, as well as to record and transcribe the session. The responses of each participant were transcribed and recorded as depicted in Supplementary Table 2. The language used was Malay and English based on the participants preferences. For interviewee who responded in Malay, translation to English were completed prior to transcribing and recording in the respective table. Below are the examples of the open-ended questions asked during the interview:

“What are the other challenges that you faced during COVID-19 pandemic as a caregiver?”

“What kind of support you wish you could have received during the pandemic while delivering care?”

### 2.4 Statistical analysis

The statistical analysis of the data in this study was performed using the IBM SPSS Statistics (Version 28). The normality of the data was explored using Kolmogorov–Smirnov test. An independent-samples t-test was used to compare caregivers' burden between the two demographic groups such as gender (male vs. female) and education level (tertiary vs. secondary). For categorical variables with more than two levels, such as race, marital status (e.g., married, single, divorced), income type, and employment status (employed, unemployed, sel-employed), a one-way analysis of variance (ANOVA) was conducted.

Pearson's correlation coeficient was performed to examine the relationships between all relevant variables. Finally, multiple regression analysis were performed to determine sociodemographic and social connection as predictors towards the burden of stroke caregivers. Given the exploratory nature of this study and the absence of strong *a priori* hypotheses regarding which specific social connection variables would best predict each domain of caregiver burden, a stepwise multiple regression approach was employed. This approach was chosen to empirically identify the most parsimonious set of predictors from the available social connection measures (social isolation, loneliness, and social support, including their respective subscales) and sociodemographic variables. To ensure stability of the estimates, multicollinearity was assessed using variance inflation factors for all retained predictors.

Two-tailed tests were used for all statistical analyses. The multiple regression analysis was performed on the caregivers' burden as an outcome variable (types of burden) against the independent variables such as social connection (social isolation, loneliness and social support). However, multiple regression on outcome variables against sociodemographic predictors was performed separately.

## 3 Results

### 3.1 Demographic profile

Table 1 summarises the demographic variables in terms of sample sizes (*n*) and percentage (%), mean (*M*) and standard deviation (*SD*). The caregiving duration was collected in days, weeks, and months, and then standardised into total months. Two missing data which from the total income, age, and caregiving duration variables were addressed using listwise deletion (27), thus leaving 92 participants only for these three variables (as seen in Table 1). This led to only 2.1% of the data was missing, which was below the threshold levels of 20% (27, 39) thus, it had no impact on the analysis of this study. For categorical variables, comparisons were made using independent *t*-tests (two-tailed) or ANOVA. It was found that caregivers with tertiary education had significantly greater caregivers' burden than caregivers with secondary education, *t* (92) = 2.65, *p* = .009, Cohen's *d* = 0.70, indicating a moderate effect size. Differences on caregivers' burden among other demographic factors were not significant (all *p*s ≥ .074).

**Table 1:** Demographic descriptives and comparison between different sociodemographic groups on stroke caregivers' burden.

Variables	*T*-test/ANOVA				
	*n* (%)	*M*	SD	*t*/*F*	*p*
Continuous					
Age	92 (98.0)	24.51	41.74	—	—
Caregiving duration (months)	92 (98.0)	37.91	10.41	—	—
Total income (monthly)	92 (98.0)	3,144.35	2,429.92	—	—
Categorical					
Caregiver type 1				0.51	0.947
Household	80 (85.1)	29.84	17.07		
Non-household	14 (14.9)	29.50	19.61		
Caregiver type 2				1.55	0.371
Family	86 (91.5)	30.28	17.68		
Non-family	8 (8.5)	24.50	13.10		
Gender				0.18	0.920
Male	27 (28.7)	30.07	17.53		
Female	67 (71.3)	29.67	17.41		
Race				0.54	0.583
Malay	80 (85.1)	29.8	16.05		
Chinese	7 (7.4)	34.57	32.75		
Others	7 (7.4)	24.86	12.28		
Marital status				2.60	0.080
Single	30 (31.9)	34.90	20.08		
Married	61 (64.9)	27.92	15.65		
Divorced	3 (3.2)	16.67	7.64		
Education level				6.32	0.009
Secondary	18 (19.1)	20.33	10.71		
Tertiary	76 (80.9)	32.03	17.92		
income type				0.23	0.922
Monthly income	68 (73.1)	32.59	17.88		
Monthly pension	5 (6.4)	24.80	12.64		
From family	16 (14.1)	27.50	16.11		
Official financial aid	1 (1.3)	27.00	—		
Multiple sources	4 (5.1)	32.25	24.35		
Employment				2.68	0.074
Unemployed	26 (27.7)	29.88	14.59		
Employed	56 (59.6)	31.96	18.81		
Self-employed	12 (12.8)	19.41	12.34		

The results for the measured variables including overall stroke caregivers' burden, social isolation, loneliness, and social support for all 94 participants were (*M*, *SD* = 29.79, 17.36), (*M*, *SD* = 12.25, 6.59), (*M*, *SD* = 5.01, 1.76, 6.59) and (*M*, *SD* = 24.10, 17.36), respectively.

Correlational analyses were conducted using Pearson Correlation tests with the cut-off point of; weak, *r* = .10 to .29, moderate, *r* = .30 to .49 and strong, *r* = .50 to 1.0 (40). All assumptions for normality were not violated based on the skewness value (41) (see Supplementary Table 1). Loneliness and caregivers' burden were found to be moderately, positively correlated, *r*(92) = .30, *p* = .004, 95% CI [0.10, 0.48], indicating that the lonelier the caregivers are, the greater the caregivers' burden. An inverse and a moderate correlation between social support and caregivers' burden was found, *r*(92) = −.34, *p* = .001, 95% CI [−0.51, −0.14], indicating that the higher the social support, the lower the caregivers' burden. The social isolation was found to be weakly and negatively correlated with the emotional burden, *r*(92) = −.21, *p* = .041, 95% CI [−0.40, −0.01] and social burden *r*(92) = −.24, *p* = .022, 95% CI [−0.42, −0.04]. This indicates that the higher the social isolation, the lower the emotional and social burden.

### 3.2 Social connection as predictors of stroke caregivers' burden

A stepwise multiple regression was used to identify the predictors towards caregiver's burden. Besides, multiple regression was also conducted using each construct of caregivers' burden as dependent variable (time burden, developmental burden, physical burden, emotional burden, and social burden). The predictors were social isolation (family isolation and friend isolation), loneliness, and social support (social interaction, subjective support). The regression coefficient results of social connections predictors are depicted in Table 2. Social support was found to significantly predict the overall caregivers' burden and emotional burden. The model summary indicates that 12% of the variations in the caregivers' burden can be explained by social support, whilst the same predictor explains 16% variance of the emotional burden. On the other hand, loneliness itself significantly predicted both developmental and physical burden, explaining 11% and 8% of the variance respectively. Furthermore, subjective support significantly predicted social burden and explains 17% variance.

**Table 2:** Regression coefficients of social connections predicting caregivers' burden and domains**.**

Predictor variable	Criterion variable	*F*	*R* ^2^	*B*	*SE B*	*Β* (95% CI)	*t*	*p*
Social support	Caregivers' burden	11.97	0.12	−1.38	0.40	−0.34 (−2.17, −0.59)	−3.46	<.001
Loneliness	Developmental burden	11.20	0.11	0.93	0.28	0.33 (0.38, 1.48)	3.35	.001
Loneliness	Physical burden	7.64	0.08	0.69	0.25	0.28 (0.19,1.18)	2.76	.007
Social support	Emotional burden	17.13	0.16	−0.36	0.09	−0.40 (−0.54, −0.19)	−4.14	<.001
Subjective support	Social burden	18.98	0.17	−0.58	0.13	−0.41 (−0.84, −0.32)	−4.36	<.001

CI, confidence interval.

**Figure 1:**
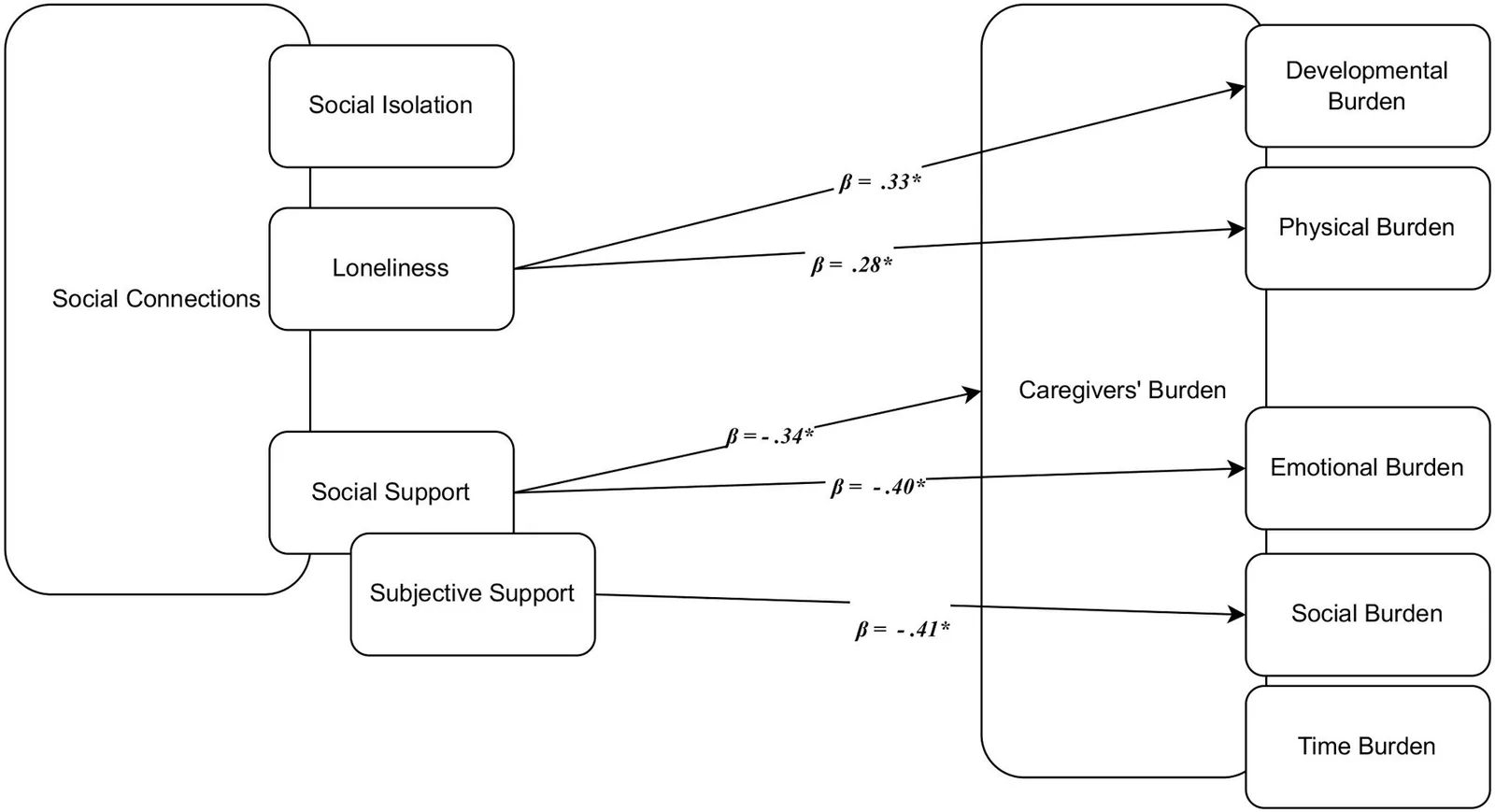
The model of social connections predicts the burden of stroke caregivers.

Figure 1 further depicts the social connection predictors of caregivers' burden in each domain.

### 3.3 Education levels as predictors of stroke caregivers' burden

Sociodemographic predictors were then analysed separately from social connection predictors and only some of the predictors were selected in the regression model using a stepwise method. This includes age, caregiving duration (in months), total income, employment and education to predict the caregivers' burden as these variables met the assumptions which correlated with caregivers' burden significantly. The education levels of stroke caregivers in this study were recoded using dummy variables where “tertiary education” is coded as 1 and “secondary education” was coded as 0 (reference dummy). Education was found to significantly predict the overall caregivers' burden (all *p*s < .006 to .037). Particularly, tertiary education significantly predicted higher burden as compared to secondary education.

On average, caregivers with tertiary education reported an overall burden score that is 11.70 higher than caregivers with secondary education (see Table 1). Table 3 indicates the model summary, with 8% (*R*^2^ ***=*** 0.08) of the variations in the caregivers' burden can be explained by the changes in education (*β* = 0.28, *t* = 2.81, *p* = .006) significantly.

**Table 3:** Regression coefficients of tertiary education predicting caregivers' burden and domains.

Predictor variable	Criterion variable	*F*	*R* ^2^	*B*	*SE B*	*Β* (95% CI)	*t*	*p*
Tertiary education	Caregivers' burden	7.92	0.08	12.76	4.54	0.28 (3.75, 21.8)	2.81	.006
Tertiary education	Developmental burden	6.08	0.06	3.23	1.31	0.25 (0.63, 5.83)	2.47	.016
Tertiary education	Physical burden	8.34	0.09	3.30	1.14	0.29 (1.03, 5.56)	2.89	.005
Tertiary education	Emotional burden	4.49	0.05	2.20	1.04	0.22 (0.14, 4.25)	2.12	.037

CI, confidence interval.

**Figure 2:**
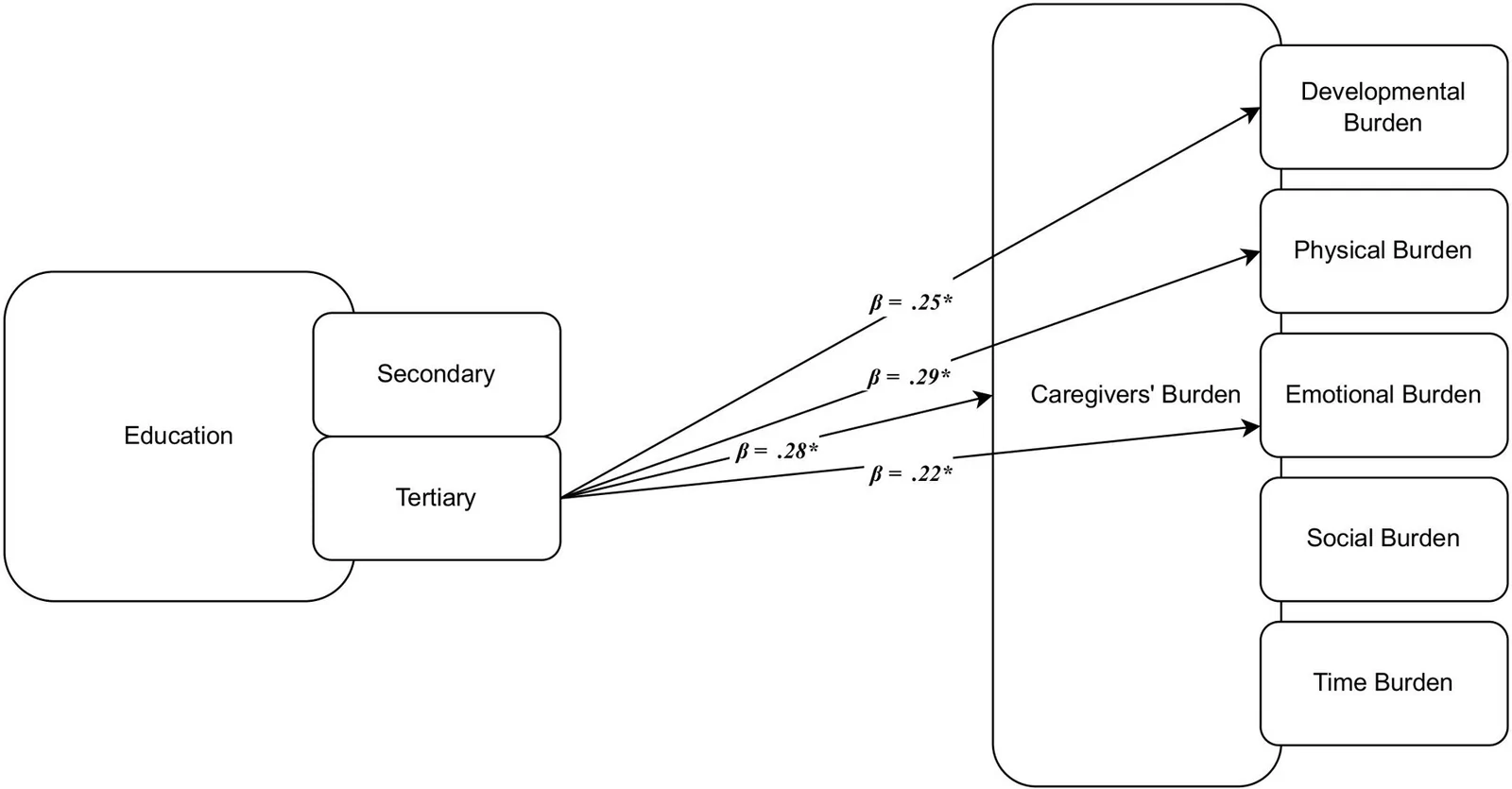
The model of tertiary education predicts the burden of stroke caregivers

The result of the regression model is significant; *F* = 7.92, *p* = .006, indicating that the difference in the caregivers' burden observed is significantly higher by 12.76% among caregivers who poses tertiary education as compared to those with secondary education (when tertiary education = 1, secondary education = 0) (see Figure 2).

### 3.4 Open-ended response

The automatic transcription from the online platform such as Microsoft TEAMS or Google Meet were observed and recorded for each interview session. The transcription was done by watching each recording multiple times simultaneously checking the automatic version of the transcriptions until the final version completed for further contextualisation following the main questions being asked. Supplementary Table 2 presents caregivers' responses to two open-ended questions collected during the lockdown, focusing on those with the highest (*n* = 4) and the lowest (*n* = 3) burden levels. The majority had hoped to receive social support from family, friends, and the community, including healthcare personnel. Financial support was preferred by caregivers with the highest burden.

## 4 Discussion

The COVID-19 lockdown has impacted beyond health, affecting people's physical and mental wellbeing, daily routines, and social and economic life (42). This study aimed to identify how different social connection (social support, social isolation, or loneliness) and sociodemographic factors contribute differently to the various aspects of burden in informal stroke caregivers during the lockdown period in Malaysia. The findings of the current study highlighted the significant predictive value of social support (e.g., subjective social support) and loneliness towards caregivers' burden and its four domains (e.g., developmental, physical, emotional, and social). Social isolation did not significantly predict caregivers' burden. Additionally, among the different sociodemographic factors, only tertiary education significantly predicted caregivers' burden.

Social support, although accounting for a modest 12% of the overall caregiver's burden during the lockdown, has a significant impact on caregivers. This modest percentage belies its crucial role in alleviating stress and providing essential emotional and practical assistance. The importance of social support for caregivers can be better understood when considering its potential to mitigate the intense pressures faced during such challenging times. Social support is crucial for stroke caregivers as it helps to alleviate the burden of caregiving (21, 43, 44). These informal caregivers often seek assistance from their local community, which includes family, friends, and cohabitants. According to Lobo et al. (21), social support significantly improves wellbeing in stroke caregiving environments and is a significant predictor of an individual's health. Social support also predicts emotional burden in the current study, consistent with the previous literature (9, 45). The diminished social support experienced by informal caregivers of stroke patients potentially decreasing the capacity to distribute their responsibilities among others (46). In the United Kingdom, support for stroke caregivers increased by 7% between 2020 and 2021 compared to pre-pandemic levels (24). Meanwhile, in Malaysia, stroke rehabilitation units were designed to be COVID-19-free during the pandemic. This includes restricting stroke caregivers to enter and accompany their families to the rehabilitation unit, contributing to compromised health outcomes for stroke caregivers (7, 12, 47).

This study's findings also indicate that increased social support reduces social burden. The provision of social support may alleviate social burden, particularly as the extended lockdowns reduced opportunities for social interaction within caregivers' networks (48, 49). The lockdown has underscored the importance of regular therapeutic interventions and support services for individuals with motor dysfunction and stroke patients (14, 46). Experts recommend developing a caregiver-therapist network to prevent delayed care and provide social support to caregivers, including formal rehabilitation staff as part of the caregivers' support network (8, 12, 50). Therefore, social support, including frequent contact and rehabilitative therapy, can help caregivers maintain emotional and physical competency during the challenging times, alleviating further caregivers' burden (45, 50, 51).

Loneliness significantly predicted informal stroke caregivers' developmental and physical burdens in this study. During the lockdown, neglecting social needs and higher caregiving responsibilities can lead to loneliness and caregivers' burden (43). Meanwhile, engaging in social, occupational, and recreational pursuits can improve wellbeing and reduce loneliness (52). During the COVID-19 lockdown, stroke caregivers faced physical health decline due to increased caregiving responsibilities without professional assistance and community based-support services (53). This lack of support may have led to loneliness and heightened feeling of missing out of life as well as personal life burden (13, 15). Without an adequate support, the increased caregiving responsibilities coupled with restrictions due to lockdown, likely contributed to feeling of loneliness. This loneliness, in turn, could have further hindered their ability to access resources and support networks, creating a negative cycle that worsened their physical health and wellbeing (43, 51).

Despite the established relationship between caregivers' burden and social isolation (52), our study suggests that social isolation, by itself, does not predict caregivers' burden. However, findings on that higher social isolation are associated with greater emotional and social burden, were somewhat surprising. One possible explanation is that some caregivers adapt to intensive caregiving demands by deliberately reducing social interactions and prioritising continuous caregiving responsibilities (54). In such cases, social isolation may not necessarily be perceived negatively, as caregivers may have accepted or normalised their reduced social engagement. Consequently, they may not report lower emotional and social burden despite being objectively isolation. This interpretation is somewhat consistent with our findings and appeared to suggest that loneliness seems to have a stronger impact on caregivers' burden (especially for developmental and physical burden in this context) regardless of their level of social isolation. A systematic review has also identified association between informal caregivers and higher loneliness levels but not social isolation (55). This is especially relevant during the COVID-19 lockdown when social isolation was strictly enforced. In another word, socially isolated caregivers who also experienced loneliness are more impacted in this context, instead of being merely socially isolated. Therefore, recognising the distinct impact of loneliness on caregivers' burden, especially during times of enforced isolation, is crucial for developing targeted interventions that effectively support caregivers' wellbeing and resilience.

In this study, the informal caregivers with tertiary education reported to experience a higher developmental burden compared to those with secondary education. It has been suggested that caregivers with higher education levels may have a better understanding of stroke rehabilitation (21) and the importance of maximising critical recovery in the first three months after stroke onset (14). However, higher educated stroke caregivers also commonly associated with a healthy lifestyle habit and promotion (56). Their concern on the risks of not using this recovery period in the pandemic mitigation, might have led to the heightened worries in the caregiving process in this study (8, 11).

Additionally, the observation that tertiary education, but not secondary education, significantly contributed to the developmental burden reported in this study may reflect differences in role expectations and opportunity costs associated with caregiving. Developmental burden refers to the perception that caregiving interferes with age-appropriate life goals, personal development, and future plans (13). Individuals with higher educational attainment are more likely to be employed, pursue career advancement, and engage in a wider range of social and recreational activities, all of which may be disrupted by intensive caregiving responsibilities (23, 57). Consistent with this interpretation, 60.6% of caregivers with the tertiary education in the current study were employed (*n* = 57). The extended lockdown and increased caregiving demands during the pandemic may therefore have heightened the perceptions of missed opportunities and role restriction among these caregivers. Besides, highly educated individuals may have greater expectations regarding personal achievement, career progression, and life planning. When caregiving responsibilities impede these goals, the discrepancy between expected and actual life trajectories may contribute to greater developmental burden. Previous studies have also reported higher levels of stress and psychological distress among caregivers with higher educational attainment, potentially reflecting greater awareness of caregiving challenges and competing occupational and family demands (58, 59). This may also explain the significant contribution of tertiary education to emotional burden in the current study. From this perspective, the association between tertiary education and developmental burden may be better understood as the consequence of increased role conflict, opportunity costs, and their associated emotional distress, rather than educational attainment itself.

### 4.1 Implication of the study

This study emphasises the importance of social support and loneliness as key factors for informal stroke caregivers' wellbeing with regard to their caregiving burden, and highlights the unique lockdown challenges faced by tertiary-educated informal caregivers. Healthcare providers and policymakers must recognise the critical role of addressing social support and loneliness needs among these caregivers, especially during another similar crises in the future. Early screening of caregivers' burden allows caregivers to be aware of their own wellbeing while committing to their caregiving duties. Providing educational courses in the healthcare system and community-based organisation to stroke caregivers could potentially enhance their social support and facilitate positive adaptation in times of crisis. Additionally, the study underscores the essential role of readily available community and social support programmes for informal stroke caregivers, fostering a culture of help-seeking to empower them and ensure they feel supported throughout their caregiving experience.

This study also reveals a previously unexplored findings—the dynamic influence of education level on caregivers' burden. While other studies have not necessarily focused on this aspect, our findings suggest that informal caregivers with higher levels of education (e.g., tertiary education) were disproportionately affected by this factor within the context of a lockdown potentially due to their heightened worries and varied lifestyles. This highlights the need for further exploration of how diverse sociodemographic factors, including education level, can interact with challenging circumstances to influence informal caregivers' experiences.

### 4.2 Study limitations

The participants on this study were purposively sampled via Facebook Stroke Survivor Malaysia Page. While this approach facilitated access to stroke caregivers, it may have introduced sampling bias. Specifically, caregivers who are active on social media are more likely to possess higher levels of digital literacy, educational attainment, and internet accessibility than those who are not engaged on such platforms (60). Sampling bias is a crucial concern in social media-based research, as it may introduce self-selection bias where individuals who choose to participate differ systematically from those who do not, resulting in a sample that may not be representative of the general population (61–63). In addition, informal stroke caregivers might have some bias when recalling the experiences of caregiving during the lockdown, due to the movement restrictions were eased later in the period. However, this bias was likely minimised as the extended and intensified pandemic lockdowns have had a substantial impact on daily life, particularly for caregivers (53).

Besides, the study did not achieve the targeted sample size, therefore reducing generalisability. The *post-hoc* G power analysis (64) shown that, in a linear multiple regression analysis with three predictors, this study was adequately powered to only detect a medium effect size (Cohen's *f*^2^ = 0.15) (64) with an 88% chance of detecting it, instead of the intended effect size with Cohen's *f*^2^ = 0.35. However, sensitivity G power analysis was conducted with the current sample size (*n* = 94) yielding a good sensitivity outcomes for this study to detect medium effect size (*f*^2^ = 0.19) with 95% (1 − *β* = 0.95) power, suggesting that our current sample size is still sensitive to finding a significant effect (32).

### 4.3 Conclusion

The social support, loneliness, and education level were indicated as key factors impacting informal stroke caregivers' burden during the lockdown, with social support being the strongest predictor. This is also supported by open-ended responses from participants which highlight the critical need for social support while caring for stroke patients. Beyond the specific context of the lockdown, our findings also suggest that individuals with higher levels of education may not necessarily more adaptable in the time of crisis when challenged by the caregiving responsibilities. This insight could inform the development of more comprehensive and tailored support programmes for all caregivers, irrespective of the education levels. Ultimately, early screening and targeted support programmes focused on social connections are crucial for significantly burdened informal stroke caregivers, highlighting the need for support group system for caregivers to be created in the government hospitals and rehabilitation services. This also highlights the necessity of researching more effective measures from the community and government. All together, the findings suggest that social support is the paramount factor in caregiving for stroke survivors as compared with other factors within social connections (4, 23). Healthcare providers and policymakers must recognise the crucial role of social support, loneliness, and educational attainment in addressing the unique needs of caregivers during crises like the COVID-19 pandemic. Nevertheless, the representativeness of the current findings may be constrained by the limited sample size and potential sampling bias; therefore, caution is warranted when generalizing the results to the broader population.

## Data Availability

The original contributions presented in the study are included in the article/Supplementary Material, further inquiries can be directed to the corresponding author/s.
