# Supplementary material for: Social connection and sociodemographic predictors of stroke caregivers' burden during COVID-19 pandemic lockdown
Source: Front Health Serv. 2026 Aug 12;6:1901816. doi: 10.3389/frhs.2026.1901816 (PMC13506378; doi:10.3389/frhs.2026.1901816)
Supplement: Supplementary file 1 [file Supplementaryfile1.docx]

**Supplementary Table 1.** *Correlations coefficients of the measured variables (including continuous sociodemographic).*

| ***Variables*** | ***1*** | ***2*** | ***3*** | ***4*** | ***4a*** | ***4b*** | ***5*** | ***6*** | ***6a*** | ***6b*** | ***7*** | ***7a*** | ***7b*** | ***7c*** | ***7d*** | ***7e*** |
| --- | --- | --- | --- | --- | --- | --- | --- | --- | --- | --- | --- | --- | --- | --- | --- | --- |
| ***1.*** Age | 1 | .29^**^ | -0.05 | -0.06 | -0.08 | -0.03 | -.26^*^ | 0.02 | -0.03 | 0.05 | -0.17 | -0.06 | -0.19 | -0.19 | -0.07 | -0.16 |
| ***2.*** Total income |  | 1 | -0.11 | 0.20 | 0.13 | .24^*^ | -0.05 | 0.20 | 0.19 | 0.14 | 0.01 | -0.02 | 0.06 | 0.08 | -0.03 | -0.05 |
| ***3.*** Caregiving duration, in month |  |  | 1 | -0.10 | -0.05 | -0.12 | 0.14 | -0.08 | -0.01 | -0.11 | -0.11 | -0.17 | -0.16 | -0.13 | 0.02 | 0.07 |
| ***4.*** Social isolation |  |  |  | 1 | .91^**^ | .91^**^ | -.23^*^ | .62^**^ | .68^**^ | .43^**^ | -0.14 | -0.07 | -0.06 | _ | -.21^*^ | -.24^*^ |
| ***4a.*** Family isolation |  |  |  |  | 1 | .66^**^ | -0.17 | .55^**^ | .54^**^ | .41^**^ | -0.08 | 0.02 | -0.02 | 0.06 | -0.17 | -.25^*^ |
| ***4b*.** Friends isolation |  |  |  |  |  | 1 | -.23^*^ | .58^**^ | .69^**^ | .38^**^ | -0.17 | -0.14 | -0.09 | -0.06 | -.21^*^ | -0.19 |
| ***5*.** Loneliness |  |  |  |  |  |  | 1 | -.37^**^ | -.24^*^ | -.34^**^ | .30^**^ | 0.01 | .33^**^ | .28^**^ | .31^**^ | .26^*^ |
| ***6*.** Social support |  |  |  |  |  |  |  | 1 | .69^**^ | .92^**^ | -.34^**^ | -0.05 | -.28^**^ | -.22^*^ | -.39^**^ | -.40^**^ |
| ***6a*.** Social interaction |  |  |  |  |  |  |  |  | 1 | .36^**^ | -.27^**^ | -0.19 | -.23^*^ | -.21^*^ | -.25^*^ | -0.18 |
| ***6b*.** Subjective support |  |  |  |  |  |  |  |  |  | 1 | -.27^**^ | 0.06 | -.24^*^ | -0.16 | -.37^**^ | -.40^**^ |
| ***7*.** Caregivers' burden |  |  |  |  |  |  |  |  |  |  | 1 | .62^**^ | .89^**^ | .89^**^ | .75^**^ | .79^**^ |
| ***7a*.** Time burden |  |  |  |  |  |  |  |  |  |  |  | 1 | .45^**^ | .49^**^ | 0.18 | .24^*^ |
| ***7b*.** Developmental burden |  |  |  |  |  |  |  |  |  |  |  |  | 1 | .83^**^ | .59^**^ | .61^**^ |
| ***7c*.** Physical burden |  |  |  |  |  |  |  |  |  |  |  |  |  | 1 | .56^**^ | .60^**^ |
| ***7d*.** Emotional burden |  |  |  |  |  |  |  |  |  |  |  |  |  |  |  | .70^**^ |
| ***7e*.** Social burden |  |  |  |  |  |  |  |  |  |  |  |  |  |  |  | 1 |

***** *p* < .05, *******p* < .01, Listwise, *N*=92
