# Supplementary material for: Social connection and sociodemographic predictors of stroke caregivers' burden during COVID-19 pandemic lockdown
Source: Front Health Serv. 2026 Aug 12;6:1901816. doi: 10.3389/frhs.2026.1901816 (PMC13506378; doi:10.3389/frhs.2026.1901816)
Supplement: Supplementary file 2 [file Supplementaryfile2.docx]

| **Supplementary Table 2.**  *Open-ended interview responses* | | |
| --- | --- | --- |
|  | | |
| Highest caregivers’ burden | 1. What are the other challenges that you had faced? | 2 What kind of support you wish you could have received? |
| 1 | *I think the challenges for me is financial as a caregiver during pandemic. We cannot really go, and people lost their job including a salary cut. Hence to sustain the life for a caregiver is difficult for instance to buy medicine as well as to meet the doctor.* | *I think would like to receive a financial assistance from government and hospital. This will help stroke patient and caregiver. Another support would be mental health consultation by the hospital for e.g. from psychologist/ psychiatrist.* |
| 2 | *For the stroke patients, many of them difficult to get medical assessment at the hospital. Caregiver is hardly access to the hospital including such as long que and no face-to-face between healthcare provider at hospital visit during pandemic.* | *From the Hospital or NGO. I need assistance in terms of money, medical supply and consultation from the mental health wellbeing due to stress during pandemic.* |
| 3 | *The challenges are difficult to get helps from other family members to assist in caregiving. This is due to my family members resides in different district/ location. The mitigation took place limits them to come and assist me to deliver care to stroke patient. Besides, I have to be extra vigilant to avoid getting COVID-19 virus contraction. I am afraid my stroke patient getting contracted.* | *The support is related to my challenges- I need support in delivering care for my stroke patients in daily basis such as bathing, getting food etc. I need helps to alleviate my burn out during caregiving.* |
| 6 | *I face mental and physical stress while delivering care to stroke patients since I’m delivering care to a bedridden patient. This is due to stroke patient dependency towards caregiver. It also worsens the health of older family member who also had other disease such as diabetes who assist in delivering care.* | *Healthcare accessibility including- house visit for bedridden stroke patients. This can be doing therapy at home as we caregivers has limited knowledge on how to appropriately deliver rehabilitation care to our stroke patient.* |
| Lowest Caregivers’ burden, |  |  |
| 4 | *Hard to seek care due to transportation and difficult to deliver care such as mobilise stroke patient. Since other family member who reside from other district or state unable to assist.*  *.* | *I think may needs moral support in terms of delivering care towards stroke patients and hoping that other family member will be allowed to cross border to come assist for taking turn in delivering care.* |
| 5 | *The challenges for me are to keep my-self steady and less contact with other people deliver care. I’m also afraid getting contracted with COVID-19.* | *I need an assistance for me to help doing exercising to stroke patients. I’m afraid stroke patient will fall while delivering care alone.* |
| 7 | *The challenge I face is to keep my safety while going to hospital bringing my stroke patients for a rehab.* | *I hope many rehabs place is not closed down. The visits have been reschedule many times and I hope rehab facility is available even during pandemic-19.* |
